# Refining and psychometric evaluation of the falling risk assessment tool in ophthalmology inpatients

**DOI:** 10.1002/nop2.1945

**Published:** 2023-07-17

**Authors:** Muling Li, Chunmei Li, Qinghui Huang, Hongzhen Zhou, Ling Xie, Fangni Chen, Shaoqin Lin, Juan Yang

**Affiliations:** ^1^ Department of Ophthalmology Nanfang Hospital, Southern Medical University Guangzhou China; ^2^ Department of Nursing Nanfang Hospital, Southern Medical University Guangzhou China

**Keywords:** falls, instrument development, ophthalmology inpatients, reliability, risk assessment, validity

## Abstract

**Aims:**

The aim of this study was to refine the Falling Risk Assessment Tool in Ophthalmology Inpatients (FRAT) and assess its psychometric properties.

**Design:**

A cross‐sectional design was used.

**Methods:**

A convenience sample of 730 patients in the ophthalmology department was recruited in a level A tertiary hospital in Guangdong Province from July 2021 to January 2022. Data were analysed using item analysis, interrater reliability, content validation, internal consistency reliability and exploratory factor analysis.

**Results:**

Five factors were extracted, accounting for 63.039% of the variance. The interrater reliability of the tool was 0.97. Cronbach's *α* was 0.658. The I‐CVI was 0.75–1.00, the S‐CVI/UA was 0.95 and the adjusted mean values of Kappa for indicators ranged from 0.72 to 1.00, as evaluated by the expert group. The FRAT showed satisfactory reliability and validity, and can be used to measure the fall risk assessment in ophthalmology inpatients.

**Patient or Public Contribution:**

After explaining the purpose, the patients received our fall risk assessment and answered the corresponding questionnaire questions.

## 1 INTRODUCTION

Studies have shown (Chew et al., 2010; Saftari & Kwon, 2018) that eye diseases can cause vision loss, reduce the reduced field of vision and create other visual impairments, affecting patients' mobility and orientation. Patients are at high risk of falling and brittle fracture of the hip due to their inability to accurately judge their own balance ability. This not only interferes with daily life and social participation but also delays recovery from illness, increases the risk of falling again and leads to physical disability and even death (Tang et al., 2019). During hospitalization, ophthalmology patients have a high risk of falling. A review of the domestic and foreign literature shows that there is no fall risk assessment tool for ophthalmic inpatients. Therefore, it is very important to determine the risk of falling in a timely manner and to take preventive measures and provide health education. To effectively prevent falls, the project team developed a specific fall risk assessment tool that can effectively identify risk groups and guide intervention measures while highlighting the relationship between ophthalmic diseases and falls (Yang et al., 2022).

### 1.1 Background

Falls in hospitals remain an ongoing concern despite worldwide recognition of this persistent problem (Heng et al., 2020). In‐hospital falls are common and serious adverse events in healthcare facilities and may lead to serious complications or even death, accounting for approximately 30%–40% of all reported accidents (Healey et al., 2008). In China, falls are the most common safety event in hospitals and have become an important factor affecting patient safety. Studies have shown that the incidence of falls is 1.3–11.5/1000 hospital days (Hill et al., 2015), with an incidence in the top three for nursing safety events. In addition, the 2018 National Nursing Quality Data Platform reported that in 2017, the incidence of falls in hospitalized patients was 0.054‰, and the injury rate was as high as 73.68‰ (Wu et al., 2019). Studies have shown that falls in hospitalized patients hinder patients' activities of daily life (ADL) and social participation and that adverse outcomes associated with inpatient falls include bruises and fractures, depression and anxiety, prolonged lengths of stay and even death. Some fall‐related incidents may even lead to lawsuits (Mikos et al., 2021). Falls not only cause economic and physical damage to patients and their families but also prompt negative changes to the patient's own psychology. This phenomenon is called fear of falling (Martinez‐Calderon et al., 2023).

A review of the literature found that most of the studies related to falls focused on elderly individuals (Klenk et al., 2017). Sex, age (Ek et al., 2019), fall history, visual impairment, eye disorders (VanNasdale et al., 2021), balance disorders, musculoskeletal disorders, chronic diseases (Morris & O'Riordan, 2017), mental health status (Radecki et al., 2018) and medication use (Laberge & Crizzle, 2019) are all major risk factors for falls in patients. However, the factors that lead to falls are different in different settings. The risk of falling among hospitalized ophthalmology patients is largely influenced by eye diseases and underlying comorbidities, and young and middle‐aged people have a higher risk of falling (Lee et al., 2020). Therefore, fall risk reduction with the help of monitoring and analysis systems along with nursing care improvement and patient education has become one of the most important issues in medical safety.

Falls occurred in seven patients in our department over the past 3 years; only one patient over 65 years of age required a fall assessment before admission, while six young and middle‐aged patients fell due to eye diseases. Therefore, the risk of falls among nonelderly inpatients in ophthalmology is still high.

In China, fall risk assessment scales are commonly used to assess the elderly population (Wang et al., 2012; Zhang et al., 2016; Zhou et al., 2012; Zhu et al., 2014). The assessment content pays more attention to factors such as age, impaired mobility, cognition, urinary incontinence, history of falls, specified medication classes and comorbidities (both number and type), and lacks the assessment of eye symptoms. Ophthalmology nurses cannot accurately identify the high‐risk groups among their patients, which increases the risk of falls and leads to an increase in the incidence of adverse events in the department.

A preliminary tool was developed through a literature review, analytic hierarchy process (AHP) and the Delphi method (Yang et al., 2022). The Falling Risk Assessment Tool in Ophthalmology Inpatients (FRAT) aims to assess the fall risk of adult inpatients to determine whether there is a high‐risk population for falls. The study group used the common fall risk assessment scale as a reference to analyse, summarize and collate relevant literature and invited clinical ophthalmologists to discuss the clinical significance and applicability of each fall‐influencing factor. Before the formal investigation, the preliminary tool was distributed to 22 experts from six provinces and cities in China to test the level of item comprehension, appropriateness of the font size, survey structure and item length. Through two rounds of expert consultation, the scale items were determined, including four factors (personal information, ophthalmic assessment, systemic assessment and social assessment), 16 items and 32 indicators. Finally, the index weight was determined by the analytic hierarchy process combined with the Delphi method, and each item was scored.

This tool includes not only common falls but also risk factors specific to ophthalmic patients, which are not included in other fall risk assessment tools. Because this scale is not widely used for ophthalmic inpatients, there is a lack of solid psychological measurements to verify its reliability and validity. Therefore, this study intends to use the tool to test its reliability and validity in a Chinese population.

## 2 METHODS

### 2.1 Study design and sample

#### 2.1.1 Design

**FIGURE 1:**
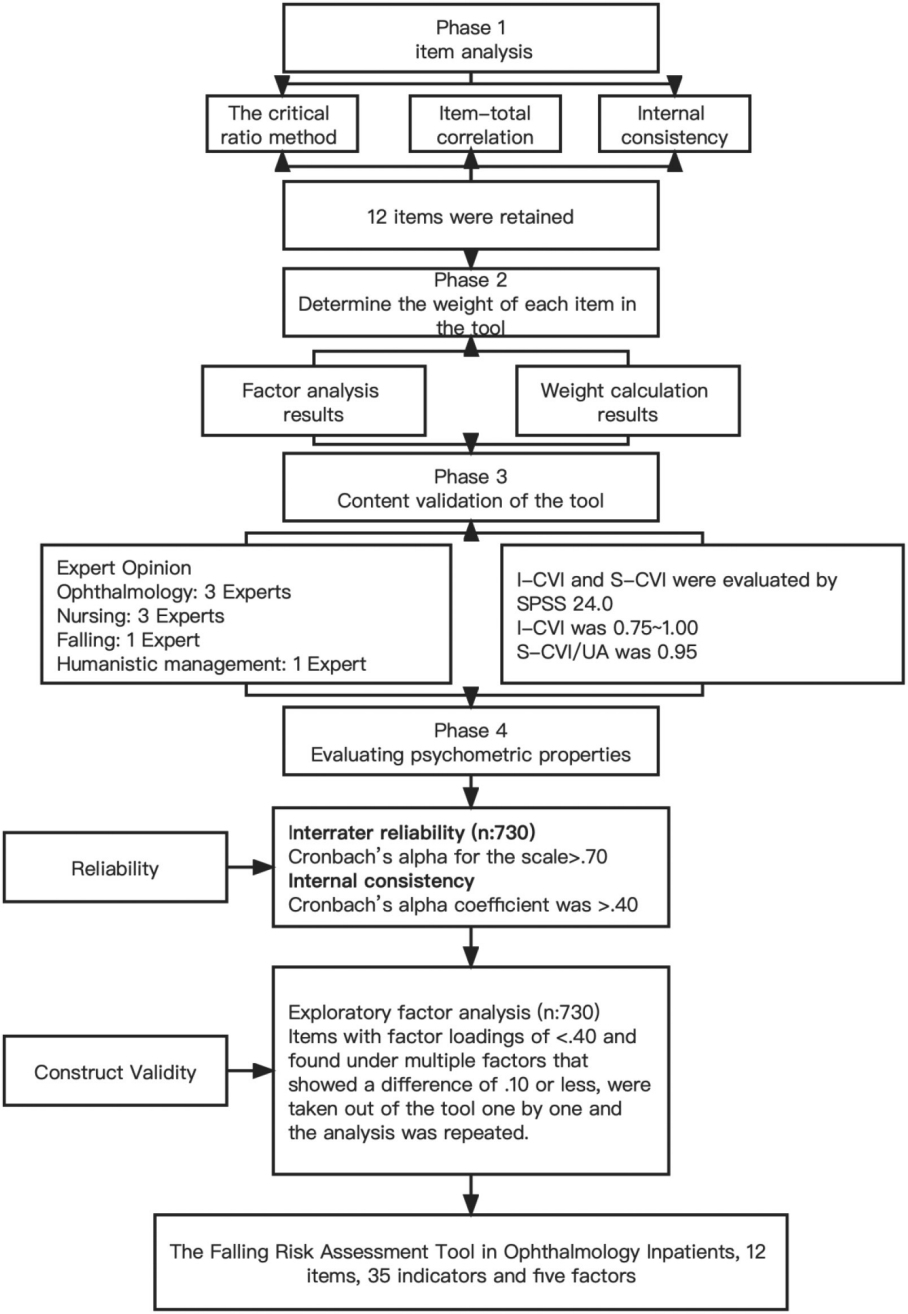
Phases in the development of the scale.

This study was performed to develop the FRAT for ophthalmology inpatients. The study included four phases (Figure 1). In phase 1, item analysis was performed. In phase 2, the weight of each item in the scale was determined. In phase 3, content validation of the tool was performed. In phase 4, the reliability and validity of the FRAT were evaluated through a cross‐sectional survey.

#### 2.1.2 Samples

*Ophthalmology inpatients.*

From July 2021 to January 2022, participants were recruited by convenience sampling among inpatients in the ophthalmology department of a tertiary hospital in Guangzhou, China, to participate in a field investigation (*n* = 730). Chinese‐speaking ophthalmology patients were eligible to participate if they (1) were 18 years of age or older, (2) were ophthalmology inpatients and (3) were willing to participate in this study.

*Experts.*

Delphi experts (*n* = 8) were invited to conduct expert evaluation for content validity analysis. The eligibility criteria for Delphi experts were a minimum of 5 years of experience in clinical ophthalmology, fall‐related research, clinical nursing and humanistic management in these fields. Experts came from three provinces in western, northern and south‐eastern China. The mean age was 50.50 ± 5.01 years. Half of the experts (50%) had doctoral degrees, and the average working time was 31.75 ± 6.00 years.

### 2.2 Ethics statement

Approval for the study and data collection was granted by the committee of Nanfang Hospital of Southern Medical University (No. NFEC‐BPE‐010). Informed written consent was obtained from the patients after the study's purpose, content and method were explained. The ability to voluntarily withdraw from the study, the protection of information and the anonymous storage of data were guaranteed.

### 2.3 Instruments of data collection

The tool was developed by the authors to assess the risk of falls in ophthalmic inpatients. It included 18 items, namely fall history, stroke history, anaemia history, chronic diseases, age, corneal irritation, double vision, visual acuity, visual field, balance ability, lower extremity sensation, excretion cases, drug use, cognition, infusion situation, caregiver, dressing and self‐competence recognition, providing a total score in the range of 9–45 (Yang et al., 2022). The sociodemographic variables and clinical data obtained from the participants included sex, age, marital status, residence, educational level, occupation and primary caregiver.

### 2.4 Data collection procedure

Three researchers were involved in the process of data collection for the field investigation, which included a field survey and an online survey. Before the study, researchers were trained on the survey content, survey skills, survey instructions and some matters needing attention. All data collected and entered were double‐checked for quality assurance. This study included four steps. Specifically, in phase 1, the item analysis method was used to select and optimize items. In phase 2, the item weights and scoring criteria were determined. In phase 3, test the content validity of the tool. In phase 4, collect the participants' data, and establish the psychological characteristics of the tool.

#### 2.4.1 Phase 1: Item analysis

To improve the sensitivity of indicators, the items were selected through the CR method, the internal consistency and the correlation coefficient method (Li et al., 2020): (1) The critical ratio method was used to rank the total scores for the tool, 27% of the sample with the highest and lowest scores were identified, and an independent samples *t* test was used to compare the scores between the two groups. Items were eliminated if there was no statistical significance (*p* > 0.05). (2) The correlation coefficient between the total score of the scale and the item was calculated, and items were eliminated if there was no statistical significance (*p* > 0.05). (3) The total correlation of each item and the Cronbach's *α* coefficient was calculated. If Cronbach's *α* coefficient was <0.3, this meant that the stability is low, and the item was considered to be deleted (Yu et al., 2022). In a comprehensive discussion, items that satisfied two or more conditions were deleted without a serious impact on the tool's structure.

#### 2.4.2 Phase 2: Determine the weight of each item in the scale

After item analysis, principal component analysis was used to calculate item weight. In this study, characteristic values, factor loading values and variance percentages were extracted by exploratory factor analysis using the same method. The load coefficient and variance contribution rate of each factor were determined, multiplied to obtain the weight, and then normalized to obtain the weight (Wang, 2019). According to the calculation result of the item weight value, each item of the scale was scored again.

#### 2.4.3 Phase 3: Content validation of the tool

In step three, the researchers gathered a panel of experts to establish evidence on the content validity of the tool. The panel consisted of eight experts in the areas of clinical ophthalmology, fall‐related research, clinical nursing and humanistic management. Panel members were experts in either clinical measurement and evaluations or experienced nurses or physicians who had worked in the Ophthalmology Department for more than 5 years and were very familiar with the clinical assessment of falls. An evaluation form was designed to guide the review process and record the comments from panel members about the scale. Closed and open‐ended questions were included in the questionnaire. Since the tool assessed the fall risks of inpatients in the Ophthalmology Department, the closed questions required the experts to determine the relevance of each item of the tool on a four‐point scale.

#### 2.4.4 Phase 4: Psychometric evaluation

The nurses of the Ophthalmology Department received training on how to rate the tool. Since the tool was primarily designed for use by healthcare professionals to evaluate patients' fall risk, it was crucial to train the raters. The training content included an explanation about the purposes of the study and the grading of the tool. To confirm their understanding of how to use the tool, the participating nurses carried out the tool ratings with the instructor. First, questions about and difficulties with the tool were identified. This was followed by a discussion of the tool until a consensus was reached. Subsequently, a field test was performed before the actual administration of the tool. The purpose of the test was to identify potential scoring difficulties in a real hospital environment. Interrater reliability was assessed to determine the reliability of the tool in one of the study hospitals. The psychometric evaluation of the tool also included the determination of internal consistency reliability and construct validity.

### 2.5 Statistical analysis

Descriptive statistical analysis, item analysis, content consistency analysis, validity analysis and exploratory factor analysis were conducted for the scale using SPSS 24.0. Interrater reliability: Correlation analysis was used to measure the correlation between the total score and items of the tool. The internal consistency of the scale was assessed using Cronbach's *α*. The content validity of the tool was determined by calculating the content of validity index (CVI), which described the extent of agreement between the eight experts. The construct validity was analysed with principal component analysis (PCA). The suitability of data for PCA was assessed using the Kaiser–Meyer–Olkin (KMO) test and Bartlett's test of sphericity. Generally, the proportion of the total variance explained by the retained factors should be at least 50% (Streiner, 1994).

## 3 RESULTS

### 3.1 Sample characteristics

For the field investigation, 730 out of 761 paper questionnaires were valid, with a recovery rate of 95.9%. The field investigation included 377 males and 353 females, with a mean age of 57.00 ± 15.26 years (see Table 1).

**TABLE 1:** Ophthalmology patients characteristics (*N* = 730).

Demographics	Field investigation		Variable	Field investigation	
	*N*	%		*N*	%
**Age**			**Marital status**		
18–30	54	7.4	Unmarried	60	8.2
31–50	182	24.9	Married	597	81.8
51–65	284	38.9	Divorced	18	2.5
>65	210	28.8	Widowed	54	7.4
**Sex**			**Medical payment**		
Female	353	48.4	Self‐pay	48	6.6
Male	377	51.6	Employee medical insurance	221	30.3
**Education level**			Urban medical insurance	275	37.7
≤Primary	183	25.1	New rural cooperative medical system	186	25.5
Middle	238	32.6	**Monthly income per person (10,000)**		
High	161	22.1	≤0.45	489	67.0
≥Bachelor's degree	148	20.2	0.45–0.8	157	21.5
**Employment status**			>0.8	84	11.5
On‐the‐job	364	49.9	**Residence**		
Retired	210	28.8	Rural	224	30.7
Unemployed	156	21.4	Sub‐rural	140	19.2
			City	140	19.2

### 3.2 Item analysis

#### 3.2.1 Critical ratio method

In this study, the high‐low total score method was used, that is, the total scores were sorted. The top 27% were divided into the low group (total score ≤ 6, 203 cases), and the bottom 73% were divided into the high group (total score ≥ 15, 207 cases). A *t* test was conducted for each item. There were no statistically significant differences in A3 Anaemia history, B1 Corneal irritation, B2 Double vision, C5 Cognition, C6 Infusion situation, D2 Dressing and D3 Self‐competence recognition (*p* > 0.05) (see Table 2).

**TABLE 2:** Critical ratio analysis result.

Items	Low group (*n* = 203)	High group (*n* = 207)	*t*	*p*	Outcome
A1 Fall history	0.04 ± 0.40	1.02 ± 1.75	−7.893	0.000	Accepted
A2 Stroke history	0.03 ± 0.24	0.41 ± 0.81	−6.425	0.000	Accepted
A3 Anaemia history	0	0	—	—	Deleted
A4 Chronic diseases	0.23 ± 0.49	3.03 ± 1.53	−25.153	0.000	Accepted
A5 Age	0.18 ± 0.57	1.97 ± 2.78	−9.080	0.000	Accepted
B1 Corneal irritation	0.33 ± 0.66	0.22 ± 0.66	1.733	0.084	Deleted
B2 Double vision	0.01 ± 0.14	0.11 ± 0.14	−1.388	0.167	Deleted
B3 Visual acuity	0.16 ± 0.68	4.12 ± 4.70	−11.982	0.000	Accepted
B4 Visual field	0.31 ± 0.79	1.86 ± 2.21	−9.480	0.000	Accepted
C1 Balance ability	0	0.77 ± 1.81	−6.173	0.000	Accepted
C2 Lower extremity sensation	0	0.06 ± 0.34	−2.480	0.014	Accepted
C3 Excretion cases	0.25 ± 0.60	1.26 ± 0.65	−16.433	0.000	Accepted
C4 Drug use	0.33 ± 0.90	3.37 ± 1.39	−26.325	0.000	Accepted
C5 Cognition	0	0	—	—	Deleted
C6 Infusion situation	0	0 ± 0.07	−1.000	0.318	Deleted
D1 Caregiver	2.07 ± 2.00	2.47 ± 1.95	−2.072	0.039	Accepted
D2 Dressing	0	0	—	—	Deleted
D3 Self‐competence recognition	0	0	—	—	Deleted

*Note*: For the three items, the standard deviation of the two groups was 0, so the *t* test was not performed.

#### 3.2.2 Correlation analysis

The correlation coefficient between the total score and each item score is shown in Table 3. Items such as A3 Anaemia history, B2 Double vision, C5 Recognition, C6 Infusion situation, D2 Dressing and D3 Self‐competence recognition were not relevant and were deleted (*p* > 0.05).

**TABLE 3:** The results of correlation for item‐total scores.

Items	*r*	*p*	Outcome
A1 Fall history	0.347	0.000	Accepted
A2 Stroke history	0.277	0.000	Accepted
A3 Anaemia history	−0.003	0.941	Deleted
A4 Chronic diseases	0.668	0.000	Accepted
A5 Age	0.410	0.000	Accepted
B1 Corneal irritation	−0.131	0.000	Accepted
B2 Double vision	0.039	0.289	Deleted
B3 Visual acuity	0.553	0.000	Accepted
B4 Visual field	0.339	0.000	Accepted
C1 Balance ability	0.285	0.000	Accepted
C2 Lower extremity sensation	0.125	0.001	Accepted
C3 Excretion cases	0.429	0.000	Accepted
C4 Drug use	0.666	0.000	Accepted
C5 Cognition	—	—	Deleted
C6 Infusion situation	0.038	0.299	Deleted
D1 Caregiver	0.111	0.003	Accepted
D2 Dressing	0.025	0.503	Deleted
D3 Self‐competence recognition	—	—	Deleted

#### 3.2.3 Internal consistency analysis

The Cronbach's *α* of the initial tool was 0.644. The results showed that the correlation coefficients of items A3 Anaemia history, B1 Corneal irritation, B2 Double vision, C2 Lower extremity sensation, C5 Cognition, C6 Infusion situation, D1 Caregiver, D2 Dressing and ‘D3 Self‐competence recognition’ were lower than 0.3, and the above items were deleted (see Table 4).

**TABLE 4:** The results of reliability analysis.

Items	*r*	Outcome
A1 Fall history	0.416	Accepted
A2 Stroke history	0.321	Accepted
A3 Anaemia history	−0.010	Deleted
A4 Chronic diseases	0.582	Accepted
A5 Age	0.555	Accepted
B1 Corneal irritation	−0.036	Deleted
B2 Double vision	0.066	Deleted
B3 Visual acuity	0.644	Accepted
B4 Visual field	0.345	Accepted
C1 Balance ability	0.393	Accepted
C2 Lower extremity sensation	0.251	Deleted
C3 Excretion cases	0.365	Accepted
C4 Drug use	0.567	Accepted
C5 Cognition	—	Deleted
C6 Infusion situation	0.028	Deleted
D1 Caregiver	0.086	Deleted
D2 Dressing	0.013	Deleted
D3 Self‐competence recognition	—	Deleted

#### 3.2.4 Item analysis results

In this study, 18 items were analysed by critical ratio, item‐total correlation and internal consistency. The items that met two or more deletion conditions were A3 Anaemia history, B1 Corneal irritation, B2 Double vision, C5 Cognition, C6 Infusion situation, D2 Dressing and D3 Self‐competence recognition. After expert opinion and group discussion, the item ‘B1 Corneal irritation sign’ was retained. Twelve items were retained.

### 3.3 Determine the weight of each item in the scale

#### 3.3.1 Factor analysis results

The Kaiser–Meyer–Olkin value was 0.643, exceeding the recommended value of 0.60, and Bartlett's test of sphericity reached statistical significance (*χ* ^2^ = 1343.916, *p* < 0.001), indicating that correlations between the items of the tool were large enough to support PCA (Kaiser, 2016). The PCA results showed that five factors were extracted, accounting for 63.039% of the total variance.

#### 3.3.2 Weight calculation results

According to the calculation result of the item weight value, each item of the tool was scored again. The scoring criteria were as follows: items with weight coefficients of <0.02 were scored 1, those with weight coefficients of 0.02–0.04 were scored 2, those with weight coefficients of 0.04–0.06 were scored 3 and those with weight coefficients of 0.06–0.08 were scored 4. Entries with weight coefficients of 0.08–0.10 were rated 5, and entries with weight coefficients of 0.10–0.12 were rated 6 (see Table 5). The scoring results are shown in Table 6.

**TABLE 5:** Weight calculation results.

Items	Factor loading coefficient	Common factor variance contribution rate	Weight	Normalized weights	Results (points)
A1 Fall history	0.800	15.182	12.146	0.085	5
A2 Stroke history	0.648	15.182	9.838	0.099	5
A4 Chronic diseases	0.858	15.021	12.889	0.083	5
A5 Age	0.814	12.182	9.916	0.092	5
B1 Corneal irritation sign	0.555	9.636	5.348	0.089	5
B3 Visual acuity	0.545	14.195	7.736	0.102	6
B4 Visual field	0.764	9.636	7.362	0.031	2
C1 Balance ability	0.729	14.195	10.348	0.084	5
C2 Lower extremity sensation	0.810	14.195	11.498	0.082	5
C3 Excretion cases	0.794	9.004	7.149	0.074	4
C4 Drug use	0.784	15.021	11.776	0.085	5
D1 Caregiver	0.423	9.004	3.809	0.095	5

**TABLE 6:** Assignment results.

Items	Indicators	Initial score	Final score
A1 Fall history	No	0	0
	Yes	4	5
A2 Stroke history	No	0	0
	Yes	2	5
A4 Chronic diseases	No	0	0
	One disease	1	5
	Two diseases	4	10
A5 Age	18–65	0	0
	65–80	2	5
	80~	8	10
B1 Corneal irritation sign	No	0	0
	Single eye photophobia, pain, frequent tears: one symptom	1	5
	Single eye photophobia, pain, frequent tears: at least two symptoms	2	10
	Eyes afraid of light, pain, frequent tears: at least one symptom	3	15
	Eyes afraid of light, pain, frequent tears: at least two symptoms	3	15
B3 Visual acuity	VA ≥ 0.3	0	0
	0.1 ≤ VA < 0.3	3	6
	0.05 ≤ VA < 0.1	7	12
	Blind (no light perception ~VA < 0.05)	16	18
B4 Visual field	No	0	0
	Unilateral visual field impairment	1	2
	Binocular visual field impairment	5	4
C1 Balance ability	Normal	0	0
	Walk slowly/with the aid of walking equipment	5	5
C2 Lower extremity sensation	Normal	0	0
	Joint pain/numbness/tremor	1	5
	Lower limb weakness/disability/stiff muscles	2	10
C3 Excretion cases	Normal	0	0
	Diarrhoea ≥ 3 times/day, frequent urination	1	4
	Diarrhoea and urination ≥ 2 times at night	2	8
C4 Drug use	No	0	0
	Using one kind of drug	1	5
	Using at least two kinds of drugs	4	10
D1 Caregiver	Having caregiver	0	0
	No caregivers	4	5

### 3.4 Reliability

#### 3.4.1 Interrater reliability

The interrater reliability of the total tool was 0.970, and the interrater reliability of the four factors of personal information, ophthalmic assessment, systemic assessment and social assessment were 0.772, 1.000, 0.995 and 1.000 respectively. The consistency range *r* between each item was 0.981–1.000, indicating that the reliability consistency between the evaluators of the scale was high. These 20 participants were not included in the sample.

#### 3.4.2 Internal consistency

The internal consistency of the tool was determined among the participants using Cronbach's alpha coefficient. The alpha coefficient for the tool was 0.658, demonstrating that all items were sufficiently correlated in assessing fall risk.

### 3.5 Validity

#### 3.5.1 Content validity

The tool was mailed to eight experts. The results showed that the CVI for the items was 0.75–1.00, and the S‐CVI/UA was 0.95 (see Table 7).

**TABLE 7:** The content validity of the tool.

Items	I‐CVI	Pc	K^+^	Outcome
A1 Fall history	1	0.004	1	Excellent
A2 Stroke history	1	0.004	1	Excellent
A4 Chronic diseases	1	0.004	1	Excellent
A5 Age	1	0.004	1	Excellent
B1 Corneal irritation sign	0.88	0.031	0.88	Excellent
B3 Visual acuity	1	0.004	1	Excellent
B4 Visual field	1	0.004	1	Excellent
C1 Balance ability	1	0.004	1	Excellent
C2 Lower extremity sensation	1	0.004	1	Excellent
C3 Excretion cases	0.75	0.109	0.72	General
C4 Drug use	1	0.004	1	Excellent
D1 Caregiver	0.75	0.109	0.72	General

#### 3.5.2 Construct validity

*Exploratory factor analysis.*

The tool was subjected to principal component analysis with a direct oblimin rotation and Kaiser normalization. The Kaiser–Meyer–Olkin value was 0.643, exceeding the recommended value of 0.60, and Bartlett's test of sphericity reached statistical significance ($\chi^{2}$ = 1343.916, *p* < 0.001), indicating that correlations between the indicators of the scale were large enough to support PCA (Kaiser, 2016). The analysis of principal components showed the presence of five factors that accounted for 63.039% of the variance in the tool. Factor 1 was labelled ‘Independent high‐risk factors’. Factor 2 was labelled ‘Past history’. Factor 3 was labelled ‘Exercise Capacity’. Factor 4 was labelled ‘Ophthalmologic signs’. Factor 5 was labelled ‘Self‐care level’ (see Table 8).

**TABLE 8:** Component rotating structure matrix.

Factors	Items	Factor 1	Factor 2	Factor 3	Factor 4	Factor 5
Independent high‐risk factors	A1 Fall history	0.800				
	A2 Stroke history	0.648				
	A5 Age	0.814				
Past history	A4 Chronic diseases		0.858			
	C4 Drug use		0.784			
Exercise capacity	B3 Visual acuity			0.545		
	C1 Balance ability			0.729		
	C2 Lower extremity sensation			0.810		
Ophthalmologic signs	B1 Corneal irritation sign				0.555	
	B4 Visual field				0.764	
Self‐care level	C3 Excretion cases					0.794
	D1 Caregiver					0.423

## 4 DISCUSSION

In the early stage, the project team determined the initial tool and the weight of each item through a literature review, Delphi method and AHP. Based on this, the project score was formulated. In this study, the structure and content of the tool were further verified through clinical patient assessment. The results showed that the tool was scientific and effective in assessing the fall risk of hospitalized patients in the Ophthalmology Department.

First, considering the research purpose and ensuring the practical feasibility of the scale, based on classical measurement theory, three methods of critical ratio, item‐total score correlation and internal consistency were used to optimize items. When secondary indicators met ≥2 deletion conditions, they were deleted. Finally, they were rearranged into five factors (independent high‐risk factors, past history, exercise capacity, ophthalmologic signs and self‐care level with 3, 2, 3, 2 and 2 items respectively). This study combined subjective and objective item evaluation methods to reduce the influence of subjective factors, which is conducive to the internal consistency and stability of the scale's practical application. The results of this study showed that seven indicators of A3 anaemia history, B1 corneal irritation sign, B2 diplopia, C5 cognitive state, C6 infusion state, D2 wear state and D3 self‐ability identification did not meet the inclusion criteria.

The reasons were as follows: (1) Anaemia history and cognitive situation are not common clinical symptoms of ophthalmology patients; their prevalence is low, and they account for a very small proportion of the entire fall risk assessment score, so they were deleted. (2) Infusion status, dressing condition and self‐ability identification were all scored 0 in this evaluation of 730 patients. The reason may be the improvement of cultural knowledge and material living standards; That is, patients who come to the hospital for medical treatment will wear comfortable, convenient clothing, with full awareness of their health and face less risk of falls. In terms of infusion status, the treatment of ophthalmology patients, surgically and with local drugs (eye drops) and laser treatment, are the main treatments, supplemented by intravenous infusion. Routine intraocular pressure reduction is performed for glaucoma and infectious diseases. However, according to expert consultation and clinical experience, most patients have shorter infusion cycles and fewer infusions (Congress of Chinese Ophthalmological Society, 2020).

Therefore, we considered deleting the above items. In the literature review, the project team found that in addition to visual impairment, inpatients in the ophthalmology department often have diplopia (Zeng et al., 2016). Double vision will causes deviations in patients' vision and increase the risk of falling. However, the clinical investigation found that there were fewer patients in our study with diplopia symptoms and a lower risk of falling, so the entry was deleted.

The item ‘Corneal irritation sign’ met two deletion criteria, but previous studies suggested that these items were risk factors for falls in hospitalized ophthalmology patients. To improve the integrity of the scale, the item was retained in the scale after discussion by clinical experts and the project team.

The methods of determining the weight of the scale include the subjective weighting method and the factor analysis method (Shan et al., 2022). In the early stage of this study, the subjective method of expert consultation was used to assign weights to the initial scale to avoid the mismatch between weights and actual importance. At the same time, to avoid the randomness of the subjective weight determination method, the factor analysis method was used to determine the weights in combination with the clinical data, which is beneficial to improve the scientificity of the weight assignment of each item on the tool.

The results showed that the normalized weights of the entries were 0.031–0.102, sorted from large to small, in the following order: B3 Visual acuity, A2 History of stroke, D1 Caregiver, A5 Age, B1 Corneal irritation sign, A1 Fall history, C4 Drug use, C1 Balance ability, A4 Chronic diseases, C2 Lower extremity sensation, C3 Excretion cases and B4 Visual field. This may reflect the different degrees of influence of various risk factors on the risk of falls for hospitalized ophthalmology patients. Visual acuity is an influencing factor that needs to be focused on and lays the foundation for the assignment of the tool.

Reliability and validity testing is an important part of tool development and is used to evaluate the reliability, stability and validity of the tool. In this study, interrater reliability and internal consistency reliability were used to test the reliability of the tool. The results of the study showed that the reliability coefficient between the two raters was 0.970, indicating that the scale has a certain stability and will not cause large differences in the assessment results due to different raters (Hart et al., 2014).

In this study, Cronbach's *α* coefficient was used to evaluate the internal consistency of the tool. Cronbach's *α* was 0.658, indicating that the internal consistency of this tool was good (Zhang et al., 2016).

In this study, content validity and construct validity were used to test the validity of the tool. Content validity was used to measure the appropriateness and conformity of the content of the tool. The composition of the scale items was the result of the combination of a literature review, expert consultation and clinical data analysis. Each of the indicators had evidence to follow; that is, it came from the literature, was recognized by experts and was screened with clinical data. It was constructed by multiple rigorous steps. Each indicator represented the overall desired evaluation content of the tool. The research results showed that the I‐CVI was between 0.750 and 1.000, and the S‐CVI/UA was 0.950, indicating that the content validity of the scale items was good (Bjerkeset et al., 2020).

The domains and theoretical constructs of an instrument can be identified by implementing an exploratory factor analysis (Zhang et al., 2016). Evidence of the validity of the tool was provided by the results of the exploratory factor analysis. In particular, the results indicated that the structure of the tool consisted of five factors that explained 63.039% of the variance (Burn & Grove).

### 4.1 Limitations

Ideally, an independent sample should be employed to validate of the factor structure (Chua et al., 2023). Thus, further empirical evidence is needed to confirm the factor structure of the FRAT tool using another independent sample. Given that the tool development is an incremental process, the criterion validity and known‐group validity of the FRAT tool that were not tested in this study can be examined in future studies.

## 5 CONCLUSIONS

The FRAT assessment instrument is an important, targeted tool to guide clinicians and nurses in identifying fall risk in ophthalmology inpatients. This tool is evidence‐based and was evaluated by experts and patients. It has been proven to be reliable and effective and has good feasibility as well as clinical application value. A prospective study is needed in the future to analyse other psychometric properties of the tool, such as its sensitivity, specificity and predictive validity. Additionally, confirmatory factor analysis could be conducted to further verify the construct validity.

## AUTHOR CONTRIBUTIONS

*Conceived and designed the experiments*: Muling Li, Juan Yang and Hongzhen Zhou. *Acquisition of data*: Chunmei Li, Qinghui Huang, Shaoqing Lin and Ling Xie. *Analysis and interpretation of data*: Muling Li and Chunmei Li. *Drafting and critical revision of manuscript*: Muling Li. *Interpretation of data and critical revision of manuscript*: Muling Li and Fangni Chen. All authors devised the focus of this study.

## FUNDING INFORMATION

This research was supported through the Medical Scientific Research Foundation of Guangdong, China (No. B2022078).

## CONFLICT OF INTEREST STATEMENT

The authors declare no conflicts of interest.

## Data Availability

The data that support the findings of this study are available from the corresponding author upon reasonable request. The data are not publicly available due to privacy or ethical restrictions.
